# Imperceptible and Disposable Humidity and Temperature Sensors with Low Environmental Footprint Enabled by Aerosol Jet Printing and Cellulose‐Based Substrates

**DOI:** 10.1002/smtd.202500506

**Published:** 2025-04-10

**Authors:** Emily Bezerra Alexandre, Daniel Corzo, Sabine Lengger, Sandro Carrara, Jürgen Kosel

**Affiliations:** ^1^ Silicon Austria Labs GmbH Europastraße 12 Villach 9524 Austria; ^2^ École Polytechnique Fédérale de Lausanne EPFL Bio/CMOS Interfaces Lab Neuchâtel CH‐2000 Switzerland

**Keywords:** aerosol jet printing, biodegradable substrates, eco‐conscious sensors, PEDOT:PSS sensors, printed electronics

## Abstract

The continuous growth of the electronics industry requires a reevaluation of traditional materials and manufacturing techniques to address the rising issue of electronic waste (e‐waste). Environmental monitoring devices, which provide valuable insights into factors such as humidity and temperature, currently rely on non‐degradable substrates and toxic metals, significantly contributing to plastic and electronic waste. Furthermore, conventional manufacturing techniques like screen printing, while effective, are limited in their ability to produce miniaturized, high‐resolution features. Here, aerosol jet printing is used to fabricate devices for humidity and temperature monitoring, enabling minimal footprint (99.75% material reduction vs other printing methods), and precise patterning of features as small as 13 µm, even on biodegradable substrates. The resistive sensor is made of biocompatible conducting polymer poly(3,4 ethylenedioxythiophene) doped with polystyrene sulfonate (PEDOT:PSS) on a biodegradable cellulose substrate. It operates efficiently within a 10–80% RH range while maintaining a high optical transmittance of 91% in the visible spectrum. Additionally, by crosslinking PEDOT:PSS with (3 Glycidyloxypropyl)Trimethoxysilane (GOPS), the sensors effectively detects changes within a temperature range of 20–50 °C. This fully printed sensor on biodegradable substrates represents a step toward next‐generation, eco‐friendly, and metal‐free solutions for environmental monitoring while minimizing ecological impact.

## Graphical abstract

This study presents eco‐friendly humidity and temperature sensors printed on biodegradable cellulose using Aerosol Jet Printing. With 99.75% material reduction and 13 µm printing resolution, the sensors use a modified PEDOT:PSS ink for efficient operation across 10–80% RH and 20–50 °C. This fully printed sensor represents a step toward sustainable, next‐generation solutions for environmental monitoring while minimizing ecological impact.

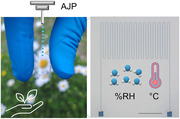

## 1 Introduction

With global attention turning toward environmental concerns, the demand for green electronics has gained prominence.^[^ ^1^, ^2^, ^3^ ^]^ Developing electronics with a reduced environmental impact requires shifting away from conventional toxic materials and resource‐intensive manufacturing processes to mitigate e‐waste.^[^ ^4^, ^5^, ^6^ ^]^ In sectors such as healthcare,^[^ ^7^, ^8^ ^]^ smart packaging,^[^ ^9^ ^]^ and agriculture,^[^ ^10^ ^]^ where disposability and cost efficiency are key, the development of eco‐conscious electronics is essential to reduce waste and resource consumption. Environmental monitoring in these fields can provide real‐time insights into individuals' health and optimize supply chains,^[^ ^11^ ^]^ all while avoiding the high costs and environmental footprint associated with recovery after usage.

Humidity sensors work by detecting the amount of moisture (water vapor) present in the air and converting it into a measurable electrical signal,^[^ ^12^ ^]^ while temperature sensors quantify changes in electrical conductivity as a function of temperature.^[^ ^11^ ^]^ Traditionally, these sensors are made with rigid materials like ceramics and metals. However, polymers offer a flexible alternative for sensor fabrication, aligning with the growing demand for adaptable and wearable electronics. Polyimide (PI),^[^ ^13^ ^]^ polypropylene (PP),^[^ ^14^ ^]^ and polyethylene terephthalate (PET)^[^ ^15^ ^]^ are commonly used substrates for these flexible sensors. Yet their loop for recycling may turn complicated due to material mixing, and during degradation, they may break down into harmful microplastic particles that infiltrate marine and terrestrial ecosystems.^[^ ^16^, ^17^ ^]^

Efforts to reduce e‐waste in environmental sensors have exploited printable materials alongside biodegradable substrates, including paper,^[^ ^18^, ^19^ ^]^ polylactide (PLA),^[^ ^20^ ^]^ and poly(vinyl alcohol) (PVA).^[^ ^21^ ^]^ When coupled with efficient additive manufacturing techniques like ink‐jet printing^[^ ^22^ ^]^ and laser‐assisted printing,^[^ ^23^ ^]^ eco‐conscious strategies can take advantage of the inherent minimized material consumption, rapid prototyping, and design flexibility. Although the printing resolution in the range of 50–100 µm^[^ ^24^ ^]^ still limits feature sizes, as deposition is dependent on drop volumes and surface energy interactions. To address this limitation, techniques like Aerosol Jet Printing (AJP) offer the potential for higher‐resolution sensors, achieving fine features down to 10 µm, and enabling the production of devices at the micron scale with different materials on various substrates.^[^ ^25^ ^]^ Moreover, AJP supports scalability with its ability to use multi‐nozzle setups for high‐throughput production, making it viable for large‐scale manufacturing.^[^ ^26^ ^]^ While AJP has been explored in eco‐conscious applications—for instance, using PVA encapsulated with polycaprolactone for temperature sensors or green graphene for humidity sensors^[^ ^27^, ^28^ ^]^—these efforts often involve multi‐step fabrication, multiple inks, and lower‐resolution features. Therefore, developing high‐resolution AJP sensors using a single ink formulation combined with biodegradable substrates like cellulose‐based materials is of significant interest. With the evolution of microelectronics, component miniaturization and imperceptibility have become pivotal for introducing advanced features into products,^[^ ^29^, ^30^, ^31^ ^]^ while reducing the overall environmental footprint is the next goal to tackle.^[^ ^32^ ^]^ Additionally, there is a growing need for transparency toward the next generation of “see‐through” electronics, particularly in smart wearables, smart glass windows, and displays.^[^ ^33^, ^34^ ^]^ Recently, Ma et al. developed a transparent humidity sensor with a fast response based on molybdenum oxide (a‐MoO3) thin films,^[^ ^35^ ^]^ while Cui et al, proposed a transparent temperature sensor based on Mn─Co─Ni─O material.^[^ ^36^ ^]^ Although these studies efficiently produced semitransparent environmental sensors, they are still constrained by the use of toxic materials and non‐biodegradable substrates, limiting their widescale adoption in healthcare, food‐safe, and agricultural environments. In addition, these sensors often require multiple materials and layers, which not only complicates the manufacturing process with extra steps but also reduces their potential for recycling.^[^ ^37^ ^]^

PEDOT:PSS is widely used as electrodes and transport layer in photovoltaics, light‐emitting devices, and bioelectronics.^[^ ^36^, ^37^, ^38^, ^39^ ^]^ It responds to environmental factors such as temperature, humidity, pressure, and strain due to its ionic conductivity and the hygroscopic nature of PSS,^[^ ^11^, ^40^ ^]^ while also being biocompatible, eco‐friendly (due to aqueous processability), and semi‐transparent.^[^ ^38^, ^41^ ^]^ Yet, miniaturizing features based on PEDOT:PSS below 20 µm remains a challenge due to incompatibility with photolithographic methods, and limitations of printing technologies (20–100 µm resolution obtained with inkjet printing).^[^ ^42^ ^]^

Here, we report on a fully printed and “see‐through” sensor based on biocompatible PEDOT:PSS printed on both rigid and flexible biodegradable cellulose diacetate substrates. We used AJP to produce high‐resolution features as small as 13, significantly smaller than those achieved by conventional inkjet or screen printing. This miniaturization also enhances the transparency of the device, with over 91% transparency in the visible range, making it nearly imperceptible. Unlike previously developed environmental platforms, this sensor uses a single ink formulation—PEDOT:PSS—to create two distinct sensor types: a humidity sensor and a temperature sensor. This versatility was enabled by crosslinking PEDOT:PSS with (3‐glycidyloxypropyl)trimethoxysilane (GOPS), allowing multifunctional use without additional complex processing steps.^[^ ^43^, ^44^ ^]^ The humidity sensor achieved high sensitivity (12.16%RH⁻¹ in the range of 10–80% RH) and demonstrated potential for real‐time health monitoring through human respiration tracking. Moreover, by incorporating GOPS into the PEDOT ink, we enhanced temperature sensitivity in the 20–50 °C range. This environmentally friendly, biocompatible, and multifunctional sensor opens up new possibilities for printed electronics with reduced material footprint.

## 2 Results and Discussion

### 2.1 Material Properties for Enhancing Humidity Sensitivity on PEDOT:PSS Blends

**Figure 1:**
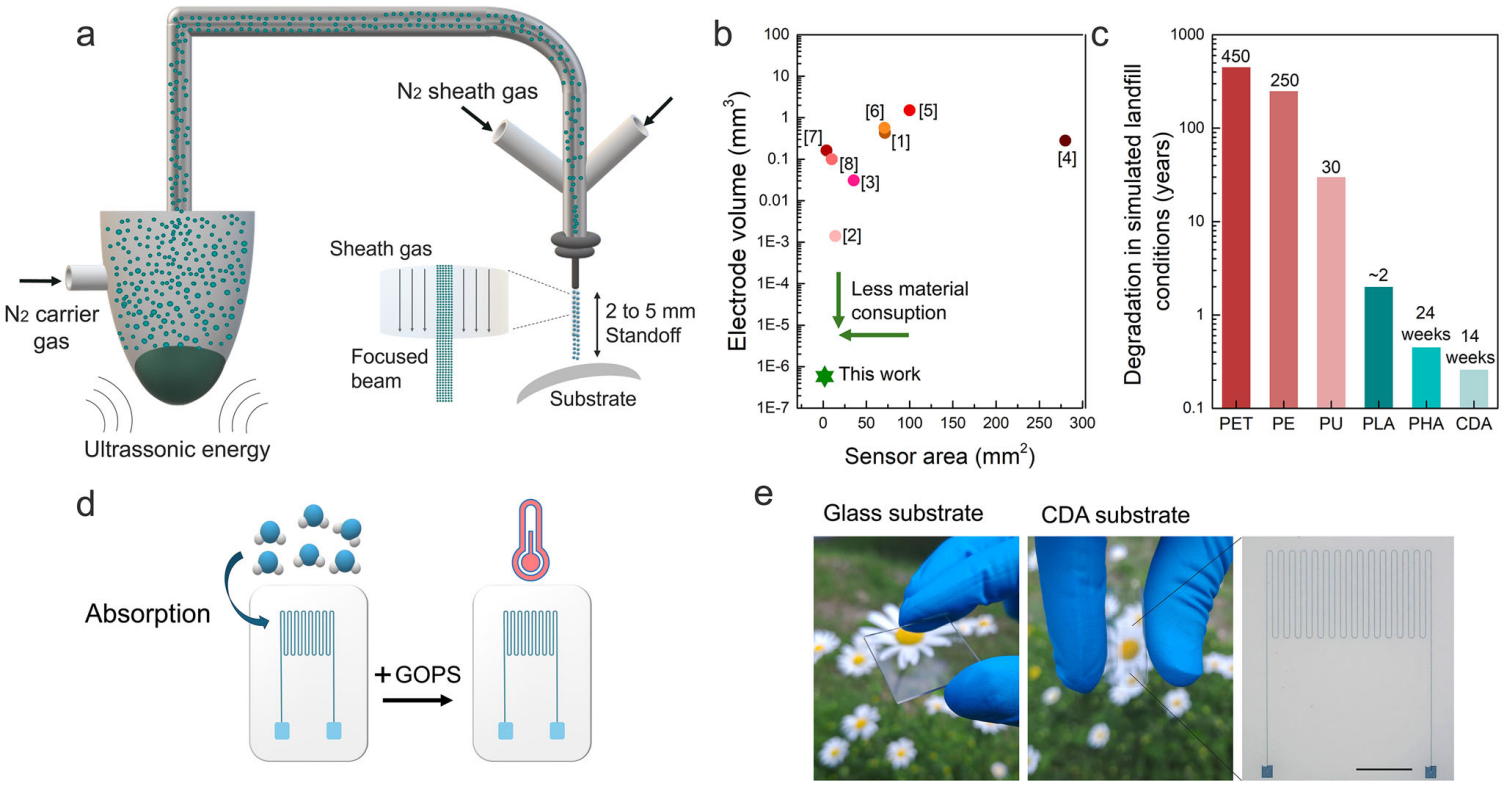
a) A schematic of the AJP process using an ultrasonic atomizer. The formulated ink undergoes ultrasonic atomization, propelled by inert gas (N2) pressure. The resulting aerosol, transported to the deposition head via a carrier gas, is focused and accelerated by an additional inert sheath gas. The jet is deposited onto the substrate through the nozzle, with the automated stage producing the desired pattern. b) Electrode volume versus sensor area of reported printed humidity sensor. c) Graph comparison of the required time in years to degrade petroleum‐based plastics and biobased plastics in simulated landfill conditions (industrial composting). d) Schematic of the sensor platform concept for humidity and temperature monitoring. e) Picture of the sensors on both glass and cellulose substrates showing a high transparency of the disposable sensor. Scale bar 2000 µm.

**Figure** **1**a denotes the proposed humidity and temperature sensing devices and the AJP fabrication process diagram. AJP is contactless, as it focuses an ink beam at a stand‐off nozzle‐substrate distance of 2–5 mm to achieve high‐resolution printing. In this process, ultrasonic atomization was selected over pneumatic atomization to create the aerosol mist for its advantages in high‐resolution applications.^[^ ^25^ ^]^ We utilized a single PEDOT:PSS ink to fabricate two kinds of sensors. The humidity sensor relies on PSS absorbing water to change its electrical properties, while the PSS crosslinking with GOPS overrides this property, thus enabling a signal change from temperature only. We printed these sensors in a serpentine geometry, known for its ability to enhance mechanical stability in flexible and stretchable devices during deformation.^[^ ^44^ ^]^ The sensors presented a total length of 7.5 mm and width of 4.5 mm, resulting in an active footprint of 1.28 mm^2^ and an electrode volume of 5.84E^−7^ mm^3^. The reduced geometrical factor leads to a material savings of ≈99.75% compared to the average consumption in conventional temperature and humidity sensors made using techniques like inkjet and screen printing (Figure 1b; Table S1 Supporting Information). We printed the sensors on both glass and biodegradable cellulose‐based flexible substrates, with the latter offering a more sustainable alternative to conventional plastic substrates such as PET) and polyethylene (PE). These traditional plastics usually take hundreds of years to degrade in soil (landfills), which remain the most common and longstanding method of plastic disposal.^[^ ^45^ ^]^ By transitioning to eco‐friendly cellulose‐based substrates, which decompose significantly faster than traditional plastics, this approach holds great potential for reducing plastic waste and its environmental impact (Figure 1c; Table S2, Supporting Information). A schematic of the sensor design and the printed sensor on glass and flexible cellulose diacetate substrate are presented in Figure 1d,e, respectively.

We measured the semitransparency of the PEDOT:PSS fine lines (Figure S1, Supporting Information). The sensor's transmissivity in the visible light range (400–800 nm) exceeds 90%, attributed to the inherent PEDOT transmittance of 85% and the high‐resolution printed lines. These fine lines, with a thickness of 0.45 µm and a width under 15 µm, are nearly imperceptible to the human eye (Figures S2 and S3, Supporting Information).^[^ ^46^ ^]^ The achieved transmittance value is comparable to that reported for transparent humidity sensors.^[^ ^35^, ^47^ ^]^

**Figure 2:**
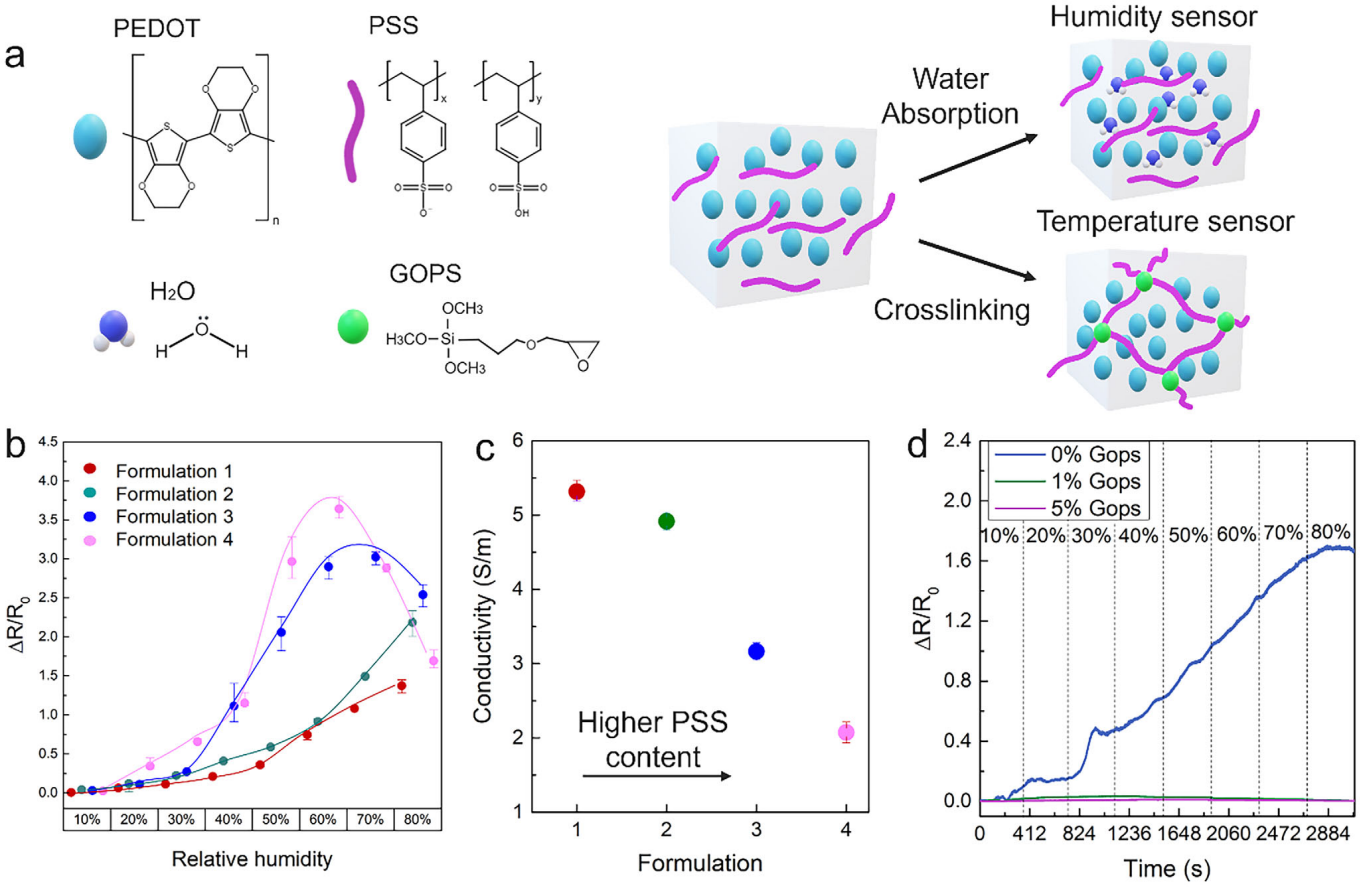
a) The sensing mechanism of PEDOT, illustrating the swelling behavior during water absorption in the humidity sensor and the crosslinking of PSS with GOPS for enhanced temperature sensitivity in the temperature sensor. b) Relative resistance versus relative humidity of PEDOT:PSS humidity sensors (*n* = 4) printed on glass slides. c) Conductivity as a function of PEDOT:PSS formulations of PH 100 and AI 4083 at 45 RH%. d) Humidity‐dependent relative resistance changes of the PEDOT:PSS AJP printed sensor on cellulose film with different GOPS concentrations.

PEDOT:PSS is a polyelectrolyte complex in which conducting PEDOT‐domains are surrounded by insulating PSS‐enriched shells.^[^ ^46^ ^]^ The electrical resistance of a PEDOT:PSS film is influenced by environmental humidity, attributed to the hydrophilic and hygroscopic nature of PSS.^[^ ^48^ ^]^ In the presence of water vapor, H_2_O molecules adsorb directly onto PSS sulfonic acid groups: *H*_2_ *O* + *PSS*(*HSO*_3_) → *H*_3_ *O* ^+^ + *PSS*(*SO*_3_)^−^,^[^ ^49^ ^]^ causing material swelling and an increase in distances between PEDOT domains, as shown in **Figure** **2**a. This, in turn, leads to an elevation in the resistance of the film, causing alterations in both its electrical and mechanical properties.^[^ ^50^ ^]^

Additionally, the temperature sensitivity of the PEDOT:PSS sensor is mainly due to enhanced charge carrier transport and generation under thermal stimuli.^[^ ^11^ ^]^ As the temperature rises, carrier hopping and tunneling within and between domains become more efficient, resulting in a decrease in the film's resistance.^[^ ^51^ ^]^ To further enhance this temperature sensitivity, GOPS is often incorporated. By crosslinking with the hydrophilic portion of the PSS units, GOPS increases the barrier for charge hopping, enhancing the sensor's temperature sensitivity while reducing its responsiveness to humidity,^[^ ^11^ ^]^ as shown in Figure 2a.

Commercial PEDOT:PSS aqueous dispersions differ in electrical conductivity, viscosities, and PEDOT to PSS ratios.^[^ ^52^ ^]^ PEDOT PH 1000 exhibits higher conductivity and lower PSS content, with a PEDOT ratio of 1:2.5, whereas AI 4083, with a ratio of 1:6, presents lower conductivity. To enhance the humidity response in PH 1000, we blended it with AI 4083 and fabricated sensors using four different volume ratios (**Table** **1**) to study the effect of PSS interference in the mixtures.

**Table 1:** Development of the initial PEDOT:PSS material formulation.

Formulation	PH 1000 [v/v%]	AI 4083 [v/v%]
1	100	0
2	90	10
3	80	20
4	70	30

We analyzed the dependence of the electrical resistance on the relative humidity (RH) by normalizing the resistance changes (relative resistance) to the initial value.^[^ ^47^, ^53^ ^]^ The relative resistance, was calculated as presented in Equation (1):

(1) $$\frac{\Delta R}{R_{0}}=\frac{\left(R_{final}-R_{initial}\right)}{R_{initial}}$$

where *R_final_* is the resistance at 80 RH%, and *R_initial_* is the resistance at 10 RH%. Figure 2b shows the humidity sensing performance of four PEDOT:PSS ink formulations. At room temperature, the presence of AI 4083 in the mixed ink formulation enhanced the relative resistance while increasing RH in the range of 10–60%, with more pronounced starting at RH >30%. At RH< 30%, the conductivity of PEDOT:PSS is dominated by conventional variable‐range hopping of electrons, which is negatively affected by the uptake of water.^[^ ^49^ ^]^ Exceeding 30 RH%, the hopping of protons between sulfonic acid groups starts to contribute to the total conductivity.^[^ ^49^ ^]^

At RH ≥70%, a downward trend is observed with the increase in the PSS loading (Formulations 3 and 4). Such a response can be attributed to the drastic deviation in the PEDOT distance inside the printed structure with reference to PSS concentration.^[^ ^38^ ^]^ The conductivity as a function of PSS loading is given in Figure 2c. Formulations 3 and 4, having a reduced amount of the highly conductive PH 1000, demonstrated the lowest electrical conductivity value. The alteration in the PH 1000 and AI 4083 volume ratio concentration, transitioning from 90/10% v/v to 70/30% v/v, corresponds to a conductivity shift from 5 to 2 S m^−1^. Therefore, we selected formulation 2 for the subsequent tests due to its high conductivity and a larger relative humidity range (10–80% RH), surpassing the other formulations.

The results for the temperature sensors utilizing PEDOT:PSS with varying ratios of the crosslinker GOPS printed on cellulose film are shown in Figure 2d. The crosslinking process between GOPS and the PSS units reduces charge hopping, leading to a reduced humidity sensitivity.^[^ ^11^ ^]^ As a result, changes in the sensor's resistance (ΔR/R₀) are primarily influenced by temperature, with charge carriers in the PEDOT phase dominating the transport properties. As a result, the introduction of GOPS enhanced temperature sensitivity. With 1% and 5% GOPS, the effect of humidity on the sensor's relative resistance became negligible.

### 2.2 Ink modification and AJP Parameters Optimization for High Resolution Printed Lines

**Figure 3:**
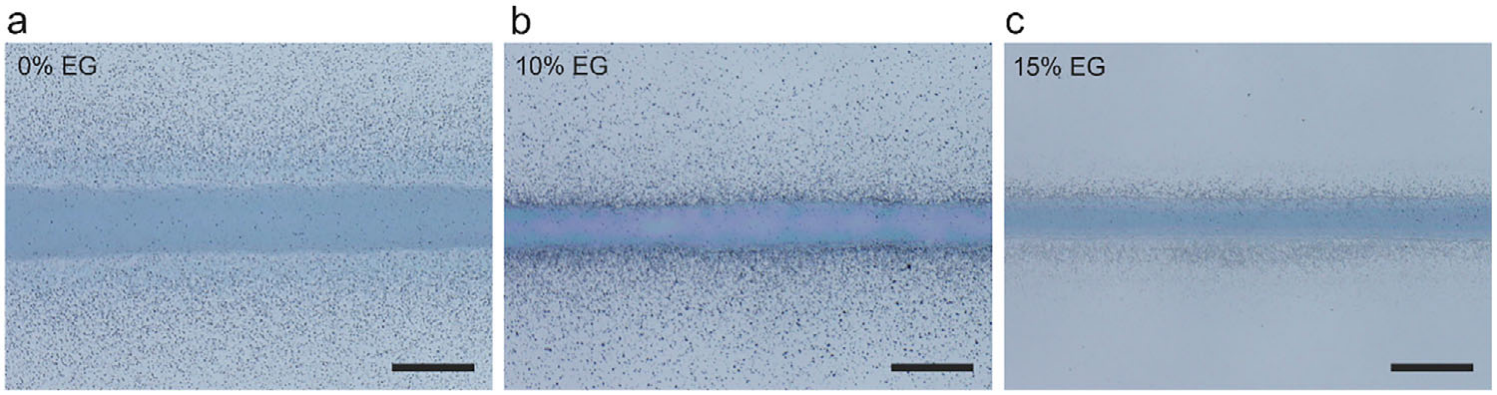
Effect of EG in ink formulation 2 on the printed lines: a) 0% v/v EG; b) 10% v/v EG; c) 15% v/v EG. Scale bar 100 µm.

Exploiting the high‐resolution capabilities of the AJP process requires reducing the production of micro droplets deposited in unintended regions on the substrate, commonly known as overspray. A pure aqueous PEDOT:PSS ink, such as that of Formulation 2, presents a high degree of overspray (**Figure** **3**a). This effect is caused by the presence of high‐volatile solvents such as water, which are normally employed to disperse PEDOT:PSS. These solvents evaporate in flight during the AJP process, and when employed independently, lead to the deposition of dry particles, contributing to overspray formation.^[^ ^25^, ^54^ ^]^

To prevent the drying of particles in the process and maintain humidity upon ink deposition, a common approach is the incorporation of 10–15% v/v of a low‐volatile co‐solvent within the ink.^[^ ^25^, ^55^ ^]^ Here we used ethylene glycol (EG) as a low‐volatile solvent, known for its efficacy in minimizing overspray in AJP co‐solvent systems.^[^ ^56^ ^]^

We tested two different EG concentrations, 10% and 15% v/v, and Figures 3b,c show how the EG‐ink modification effectively reduced undesired overspray on the printed lines. With the addition of 10% EG as a co‐solvent, a slight reduction in overspray is observed. However, the remaining overspray is likely due to low humidity and high atomization of the ink (Figure 3b). The ink consisting of 15% v/v proved as an efficient co‐solvent system for the printed lines, exhibiting a substantial overspray reduction, smooth edges, and wettability on both glass and cellulose substrates, as shown in Figures S4 and S5 (Supporting Information).

The selected co‐solvent system demonstrated the highest electrical conductivity among the alternatives, registering 88548 S/m (Table S3, Supporting Information). This result is in accordance with the effective tuning of PEDOT:PSS conductivity through the incorporation of EG.^[^ ^57^, ^58^, ^59^ ^]^ Above 15% v/v EG, a very wide line was printed, suggesting high spreading and over‐deposition of the ink, as presented in Figure S6 and Table S4 (Supporting Information).

A second requirement to produce conductive fine lines is the optimization of AJP control parameters. The focus ratio (FR) indicated in Equation (2), was identified as a crucial factor influencing line width in ultrasonic atomization.^[^ ^60^ ^]^ An increase in the focusing ratio led to a reduction in line width.^[^ ^25^, ^60^ ^]^

(2) $$Focus\;Ratio\;\left(FR\right)=\frac{Sheath\;Gas\;Flow\;Rate}{Carrier\;Gas\;Flow\;Rate}$$

**Figure 4:**
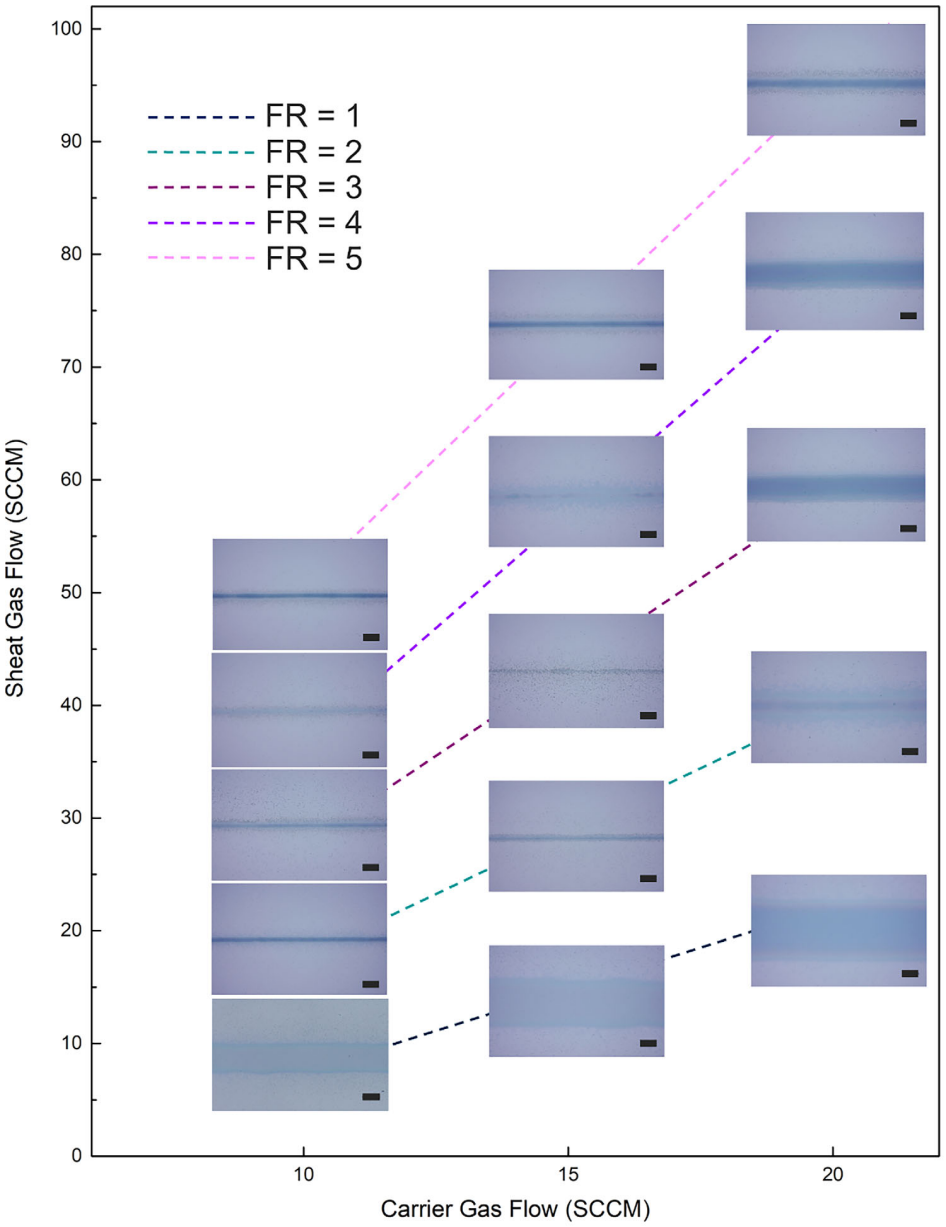
Optical microscopic images of printed lines on glass slides, illustrating the influence of sheath and carrier gas flows on the line quality. Lines printed with the same focus ratio are grouped together. Scale bar 50 µm.

The influence of the FR on the line quality is demonstrated in **Figure** **4**. A minimum atomizer flow rate of 10 SCCM had to be reached; below this value, the deposited ink is insufficient to produce a uniform line. Above 20 SCCM, the excess of deposited ink causes lines with irregular bulges; this value is in accordance with the literature.^[^ ^25^ ^]^ As illustrated in Figure 4, a decrease in the focusing ratio (FR) produced wider lines, whereas an increase in FR resulted in narrower lines. However, according to previous studies, the FR cannot be infinitely increased, as further increments no longer lead to resolution improvements and result in poorly‐defined lines.^[^ ^25^, ^61^ ^]^ In the context of our system, encompassing the ink, nozzle diameter, and printing speed, an optimal FR of 5 was found, leading to the production of uniform lines with reduced overspray.

### 2.3 Humidity and Temperature Sensor Performances

**Figure 5:**
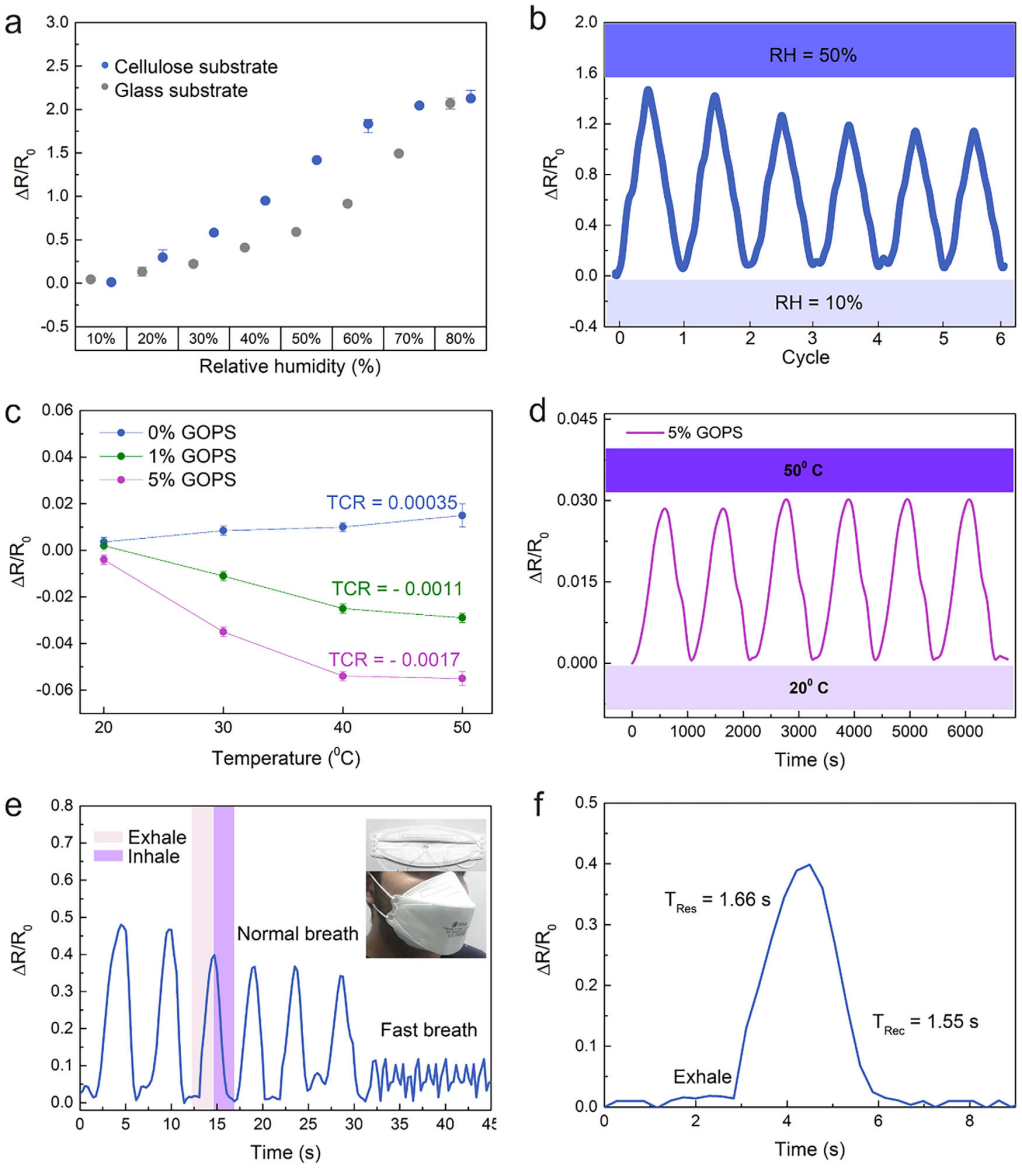
a) Relative resistance of the PEDOT:PSS humidity sensors (*n* = 4) on both glass and cellulose substrates recorded as a function of RH values at 25 °C. b) The response‐recovery curves for six cycles from 10 to 50 RH% of the PEDOT:PSS humidity sensor printed on cellulose substrate. c) Temperature‐dependent relative resistance of the PEDOT:PSS printed sensor on cellulose film with different concentrations of GOPS. d) Cyclic response of the temperature sensor with 5% GOPS printed on cellulose substrate. e) Responses of the P sensor printed onto cellulose substrate during human exhalation and inhalation under different respiratory rates. f) The zoomed in view of the highlighted curve in Figure 5e shows the response and recovery time for a normal breath rate.

We produced both temperature and humidity sensors based on a single PEDOT:PSS ink, with slight crosslinking modifications by GOPS for the temperature sensor. **Figure** **5**a shows the dynamic response of the sensor printed on both glass and cellulose‐based substrates in different humidity. As the relative humidity increased from 10% to 80% at room temperature, the relative resistance of the humidity sensor also increased on both substrates (glass and cellulose diacetate). The sensitivity (S) of the sensors is defined in Equation (3):

(3) $$S=\frac{1}{R_{i}}\frac{\left(R_{i}-R_{f}\right)}{\left(RH_{i}-RH_{f}\right)}$$

where R_i_ and R_f_ are the initial and final resistance, respectively, and RH_i_ and RH_f_ are the initial and final relative humidity. As the humidity increases, charge carrier hopping between the PEDOT chains becomes difficult, leading to an increase in resistance.^[^ ^38^, ^49^ ^]^ Consequently, we observed the highest sensitivity of 12.1609%RH^−1^ and 5.0276%RH^−1^, in the humidity range of 10–80%RH, for the sensors printed on cellulose and glass, respectively. The higher sensitivity of the sensor on cellulose film can be attributed to the increased diffusion of humidity within the substrate.

Whether during the adsorption process with increasing humidity or the desorption process with decreasing humidity, the sensitivity values of the sensor remain consistent at the corresponding relative humidity (Figure S7, Supporting Information). Figure 5b presents a six‐cycle test conducted between 10% and 50% RH, demonstrating clear and similar response‐recovery times. Notably, the curves exhibit increased stability, especially after the third cycle, suggesting the sensor's stabilization following the initial two cycles. The stability test of the AJP‐printed PEDOT:PSS humidity sensor (Figure S8, Supporting Information) demonstrates consistent performance when measuring relative humidity levels from 10% to 80% at room temperature over an 8‐day period. Moreover, measurement of the sensor after 6 months at ambient conditions denotes a linear response with a slight slope deviation, which may be attributed to corrosion and delamination of the silver electrode pads.^[^ ^62^, ^63^ ^]^

The temperature sensor performance utilizing PEDOT:PSS with the slight addition of 1% and 5% of the crosslinker printed on cellulose film is shown in Figure 5c. With a coefficient of temperature (TCR) of ‐1.7E3 for 5% GOPS addition. The repeatability of the sensor containing 5% GOPS is demonstrated with temperature values ranging from 20 to 50 °C, as presented in Figure 5d. Even with small temperature responses and without any type of passivation, the AJP printed sensor demonstrated its ability to monitor temperature.

To showcase the PEDOT:PSS sensor's suitability in wearable electronics for real‐time monitoring of human breath, the humidity sensor printed on cellulose was affixed to an FFP2 face mask, as depicted in Figure 5e, allowing continuous monitoring of human respiration. When the tester is moving slowly, the respiratory rate is normal, characterized by a “normal breath” rate. When exercising or moving fast, the respiratory rate is faster, corresponding to a “fast breath”. As a moisturizing process, exhalation increases the humidity in the sensor, while inhalation reduces the applied humidity. This aligns with our earlier humidity results, where the PEDOT:PSS humidity sensor resistance increased with a rise in humidity. The curve representing the exhalation and inhalation processes in Figure 5e is zoomed in for a closer look in Figure 5f. The response and recovery times of humidity during normal breathing are 1.66 s and 1.55 s, respectively. These values are smaller compared to other reported printed humidity sensors, as detailed in Table S5 (Supporting Information), highlighting the potential of the PEDOT:PSS humidity sensor for real‐time respiration monitoring.

## 3 Conclusion

In this study, we produced semitransparent humidity and temperature sensors with a minimal material footprint by employing AJP, utilizing a single material for both sensor types, and cellulose‐based substrates. By fine‐tuning the AJP parameters, we achieved precise control over the sensor's micro dimensions, reducing material usage and printing compact designs. The use of a modified PEDOT:PSS ink for both sensors further reduced the material consumption by minimizing the need for additional materials and extra fabrication steps. These compact sensors were printed on a biodegradable cellulose substrate, lowering their environmental impact. The PEDOT:SS material crosslinked with GOPS demonstrated minimal humidity interference, allowing its adaptation as an imperceptible temperature sensor. This adaptable platform marks a step forward in developing wearable and disposable devices with potential applications in smart packaging and transparent electronics, while contributing to reduced electronic waste.

## 4 Experimental Section

### Materials and Ink Preparation

Aqueous PEDOT:PSS PH1000 and PEDOT:PSS AI 4083 were both purchased from Heraeus, CLEVIOS. Ethylene Glycol (anhydrous, 99.8%) and (3‐glycidyloxypropyl)trimethoxysilane were purchased from Sigma–Aldrich. Cellulose‐based diacetate biofilm certified as compostable in industrial composting environments was purchased from Bleher Folientechnik GmbH. For the initial determination of a suitable print formulation, a PEDOT:PSS blend formulation was conducted with PEDOT:PSS PH1000 as the main ink and PEDOT:PSS AI 4083 as the variation ink. Four different PH1000:AI 4083 had been tested: 100/0% v/v, 90/10% v/v, 80/20% v/v, 70/30% v/v. Blend concentration was fixed at 3 mL. To further achieve a fine line resolution, a co‐solvent system test was performed. Three different PEDOT:PSS blends with incorporation of ethylene glycol (EG), Blend/EG formulations have been tested: 100/0% v/v, 90/10% v/v, and 85/15% v/v. To achieve a stable ink for microdeposition, the blend was sonicated in an ultrasound bath for 30 min prior to any deposition.

### Aerosol‐Jet Micro Deposition Process

A 5‐axis Optomec Aerosol Jet Printer controlled through a control code (G‐Code) was used to fabricate the features and devices. The prepared PEDOT:PSS inks were processed in the ultrasonic atomizer of the aerosol‐jet printer. Nitrogen was used as the inert sheath (40 sccm) and atomizer gas (40 sccm) (sccm = standard cubic centimeters per minute). A 100 µm nozzle, a scanning speed of 1.5 mm s^−1^ and a working distance of 3 mm were used throughout the process. Other machine processing parameters that were varied as part of the investigation included carrier gas flow rate (10, 15, 20 SCCM) and sheath gas flow rate (10, 20, 30, 40, 50, 60, 75, 80, and 95 SCCM). Gas flow rates were quoted as standard cubic centimeters per minute (SCCM). Following printing, the samples were cured in a conventional oven at 140 °C for 10 min. The test pattern for the high‐resolution printed lines consisted of straight lines 10 mm long. A single deposition pass was used for all prints. Glass slides and Cellulose film were used as printing substrates. Prior to printing, all the substrates were conducted to ozone plasma for 30 min. During the aerosol‐jet printing process, the substrates were kept at 40 °C. The geometry of the printed sensors was designed using AutoCAD 2018, ensuring precision and accuracy in the fabrication process.

### Morphologic Characterization of the Substrates and the Printed Features

For efficient observation and characterization of the printed lines, glass slides were chosen as the substrates for morphological characterization. For a preliminary investigation of the printed lines, optical images were taken with a VHX‐7000 digital microscope (Keyence, Belgium). The thickness of the lines was measured using a profilometer (Carl Zeiss Surfcom Touch). The contact angle of PEDOT:PSS ink on both glass slides and cellulose substrate was performed with a DataPhysics OCA 200 by dropping 3 µl of the solvent on top of the substrates. The electrode volume was calculated by multiplying the sensor‘s active area with the sensor thickness.

### Device Characterization

For humidity measurements, the sensors were physically contacted with cables and exposed to a relative humidity from 10 to 80% in steps of 10% at 20 °C, in an environmental testing chamber (Voetsch VC3 4018, Germany). The sensor sample size measured in Figures 2b and 5a was 4 (*n* = 4). The change in resistance was measured using a Keithley digital multimeter (Keithley 2700, Tektronix, Inc., USA). For the temperature measurement, the sensors were in contact with cables and exposed to temperatures from 20 to 50 °C at 40% RH. For the breath monitoring, the humidity sensor was added to a mask and measured respiration in real‐time. The transmittance of the device was tested by a spectrophotometer (Mapada V‐1600 PC). Stability tests were conducted by measuring the sensor's response to humidity (10–80% RH in 10% increments at 20 °C) daily over a period of 8 days, with measurements performed in the same environmental chamber and recorded using the Keithley multimeter. For long‐term stability tests, the humidity response (10–80% RH) of the sensor was assessed six months after production under the same conditions. Following use, the printed sensors were stored in cleanroom conditions (ISO 5) at ≈20 °C, 30% relative humidity, and 300–700 lux illumination.

## Conflict of Interest

The authors declare no conflict of interest.

## Supporting information



Supporting Information

## Data Availability

The data that support the findings of this study are available on request from the corresponding author. The data are not publicly available due to privacy or ethical restrictions.
