# Supplementary material for: Imperceptible and Disposable Humidity and Temperature Sensors with Low Environmental Footprint Enabled by Aerosol Jet Printing and Cellulose‐Based Substrates
Source: Small Methods. 2025 Apr 10;10(2):2500506. doi: 10.1002/smtd.202500506 (PMC12825335; doi:10.1002/smtd.202500506)
Supplement: Supplementary file 1 — Supporting Information [file SMTD-10-2500506-s001.docx]

**Supporting Information**

Imperceptible and disposable humidity and temperature sensors with low environmental footprint enabled by aerosol jet printing and cellulose-based substrates

Emily Bezerra Alexandre^1,2*^, Daniel Corzo^1^, Sabine Lengger^1,^ Sandro Carrara^2^ and Jürgen Kosel^1^

^1^ Silicon Austria Labs GmbH, Europastraße 12, Villach, 9524 Austria.

^2^ École Polytechnique Fédérale de Lausanne, EPFL, Bio/CMOS Interfaces Lab, CH-2000 Neuchâtel, Switzerland.

Corresponding Author

*E-mail: emily.bezerra@silicon-austria.com

Table of Contents

**Supplementary Tables**

[**Table S1.** Environmental Sensors reported on the literature in terms of sensor area and electrode volume. 2](#_Toc179280993)

[**Table S2.** Estimated decomposition of various plastics in simulated landfill condition (industrial composting). 2](#_Toc179280994)

[**Table S3.** Effect of ethylene glycol co-solvent on electrical conductivity. 2](#_Toc179280995)

[**Table S4.** Observations of print formulations utilized during initial material screen. 2](#_Toc179280996)

[**Table S5.** Biodegradable humidity sensors reported on the literature. 3](#_Toc179280997)

**Supplementary Figures**

[**Figure S1.** a) NIR–UV–vis transmittance of the printed PEDOT:PSS humidity sensor. Inset: Image demonstrating the semitransparency of the fully printed sensor. b) UV absorption spectra of single layer PEDOT:PSS humidity sensor. 2](#_Toc179277102)

[**Figure S2.** The serpentine pattern used for transmittance measurement. Only the serpentine area was used for transmittance test. 2](#_Toc179277103)

[**Figure S3.** Print formulations F1-F4 utilised during initial material screen. Nozzle size = 100μm, sheath and atomiser gas flow rates = 40 sccm, nozzle height = 3mm, stage speed = 1.5mm/s. sccm = standard cubic centimetres per minute. 3](#_Toc179277104)

[**Figure S4.** Contact angle on glass and cellulose of the PEDOT:PSS blend containing 15% v/v of EG. 3](#_Toc179277105)

[**Figure S5.** Effect of 20% v/v of ethylene glycol on the printed line. 4](#_Toc179277106)

[**Figure S6.** The dynamic sensor response at different RH of the PEDOT:PSS printed onto cellulose substrate. 5](#_Toc179277107)

**Table S1.** Environmental Sensors reported on the literature in terms of sensor area and electrode volume.

| Sensor area (mm^2^) | Electrode volume (mm^3^) | References |
| --- | --- | --- |
| 71.6 | 0.43 | [1] |
| 14.04 | 0.0014 | [2] |
| 35.52 | 0.031 | [3] |
| 280 | 0.28 | [4] |
| 100 | 1.51 | [5] |
| 71 | 0.57 | [6] |
| 4.05 | 0.162 | [7] |
| 10 | 0.1 | [8] |
| 1.285 | 5.84E-7 | This work |

**Table S2.** Estimated decomposition of various plastics in simulated landfill condition (industrial composting).

| Plastic | Reference |
| --- | --- |
| PET | [9], [10] |
| PE | [11] |
| PLA | [12] |
| PHA | [13] |
| CDA | [14] |

**Table S3.** Effect of ethylene glycol co-solvent on electrical conductivity.

| **PEDOT:PSS concentration (v/v%)** | **Ethylene glycol concentration (v/v%)** | **Electrical Conductivity (S/m)** |
| --- | --- | --- |
| 100 | 0 | 6,912 |
| 90 | 10 | 52,711 |
| 85 | 15 | 88,548 |

**Table S4.** Observations of print formulations utilized during initial material screen.

| **Formulation** | **Observations** |
| --- | --- |
| 0% EG | Complete line. Overspray distinguishable on edges of lines. |
| 10% EG | Complete line. Significant overspray. |
| 15% EG | Complete line and homogeneous. Significant reduce of overspray. |
| 20% EG | Material has spread on substrate causing a very wide line, possibly due to over deposition of the ink caused by higher humidity of the ink. |

**Table S5.** Biodegradable humidity sensors reported on the literature.

| Electrodes | Fabrication Method | Measurement range (%RH) | Substrate | Response/recovery time (s) | References |
| --- | --- | --- | --- | --- | --- |
| Silver | Inkjet Printing | 15-90 | PLA/Starch/Paper | >1/38/21 and 3/13/13 | [15] |
| Carbon | Screen Printing/Drop Coating | 35-65 | Shellac/Paper | 100.5/1297.6 and 12/789 | [1] |
| Silver | Inkjet Printing | 40-100 | Paper | 5min/4min | [16] |
| MoS2-flakes | Aerosol Jet Printing | 10-95 | Glass | 8-22 | [17] |
| PEDOT:PSS | Aerosol Jet Printing | 0-80 | Celulose-based bio film | 1.66/1.55 | This work |


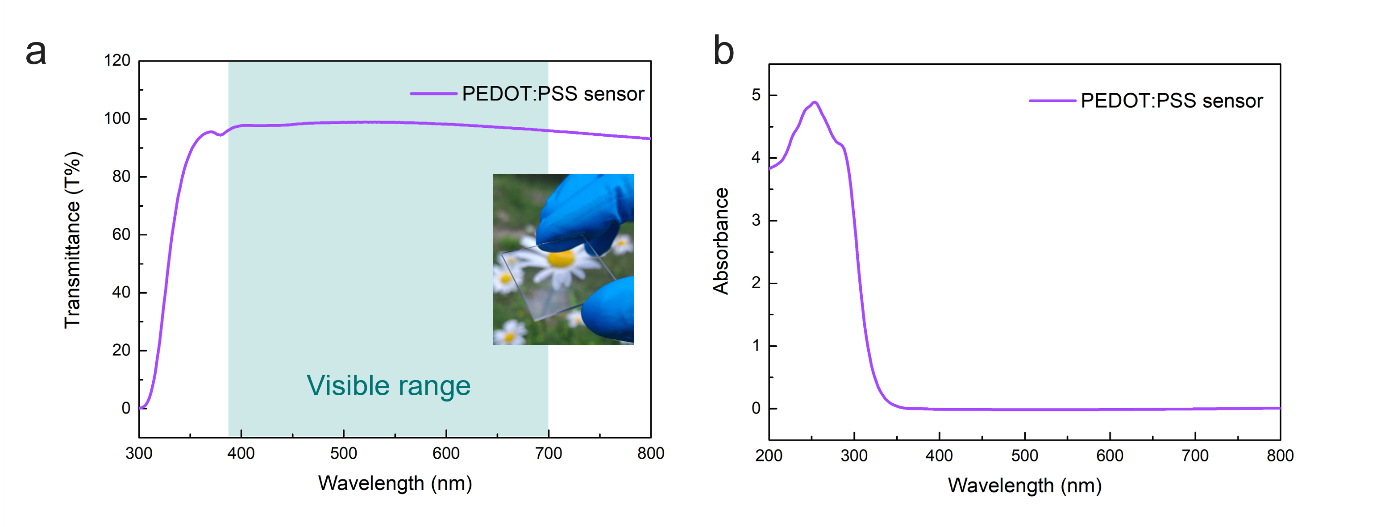


**Figure S1.** a) NIR–UV–vis transmittance of the printed PEDOT:PSS humidity sensor. Inset: Image demonstrating the semitransparency of the fully printed sensor. b) UV absorption spectra of single layer PEDOT:PSS humidity sensor.


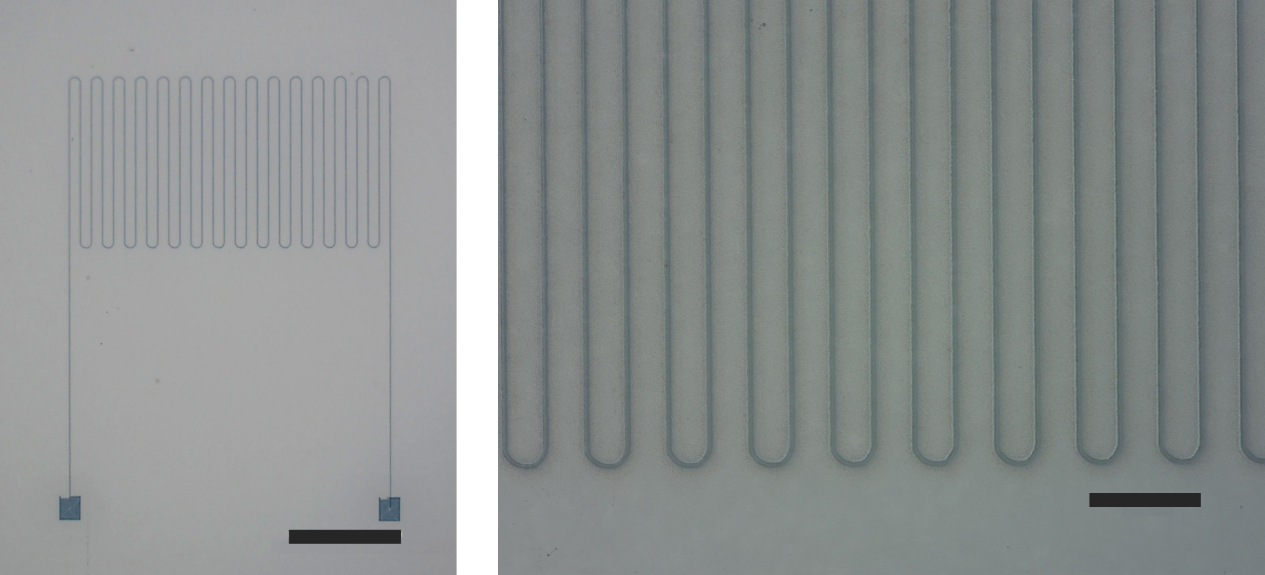


**Figure S2.** a) The serpentine pattern used for transmittance measurement. Scale bar 2000 µm. b) Only the serpentine area was used for transmittance test. Scale bar 500 µm.


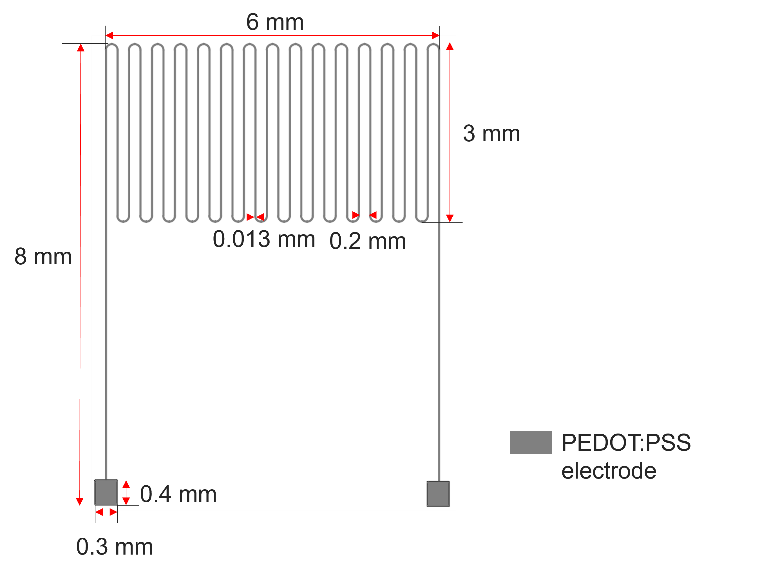


**Figure S3.** The geometry of the Aerosol Jet Printed sensor.


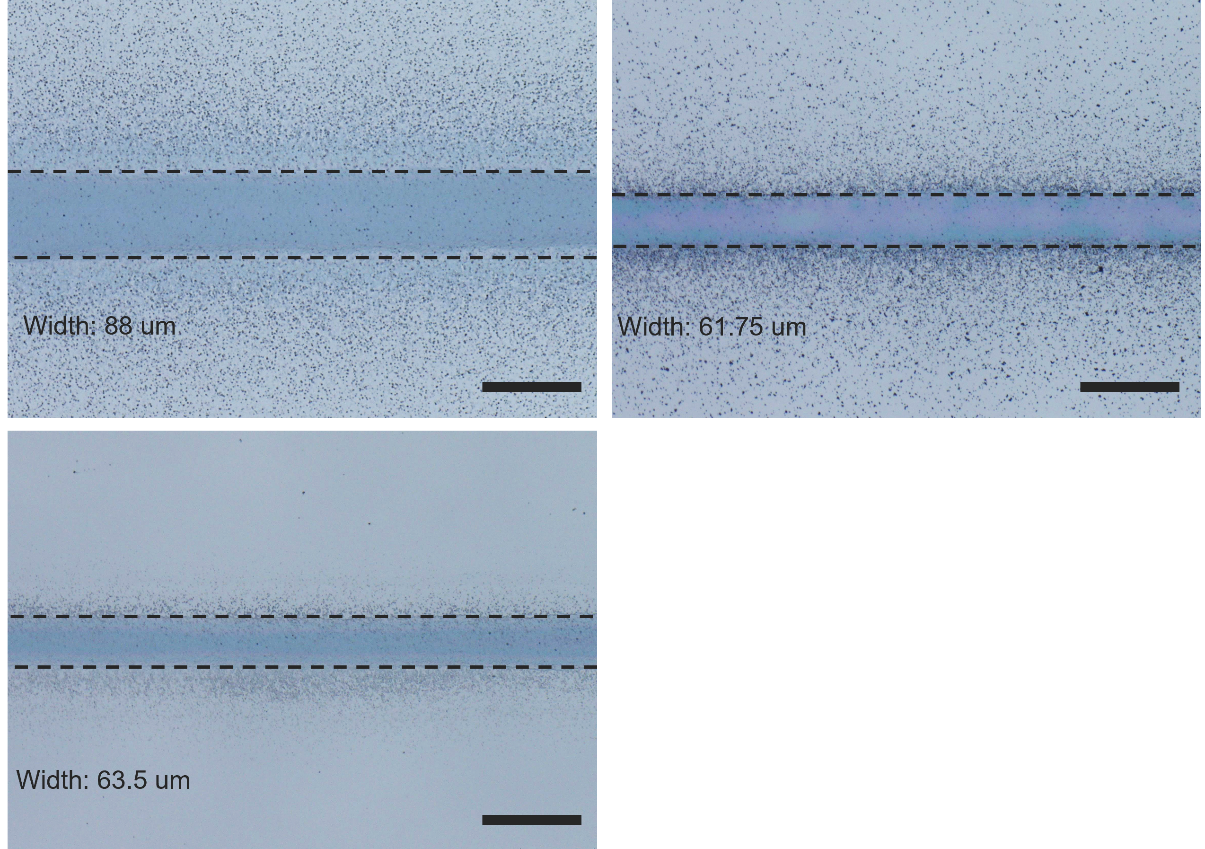


**Figure S4.** Print formulations F1-F4 utilized during initial material screen. Nozzle size = 100μm, sheath and atomizer gas flow rates = 40 sccm, nozzle height = 3mm, stage speed = 1.5mm/s. sccm = standard cubic centimeters per minute. Scale bars 100 µm.


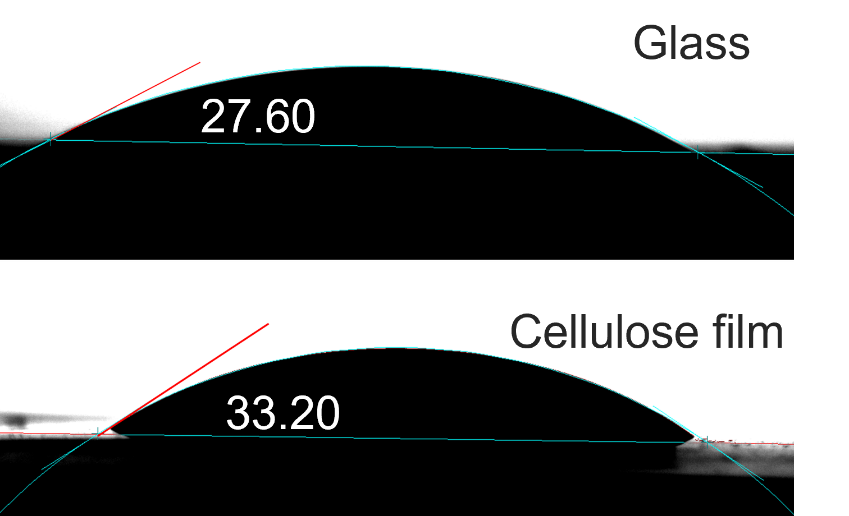


**Figure S5.** Contact angle on glass and cellulose of the PEDOT:PSS blend containing 15% v/v of EG.


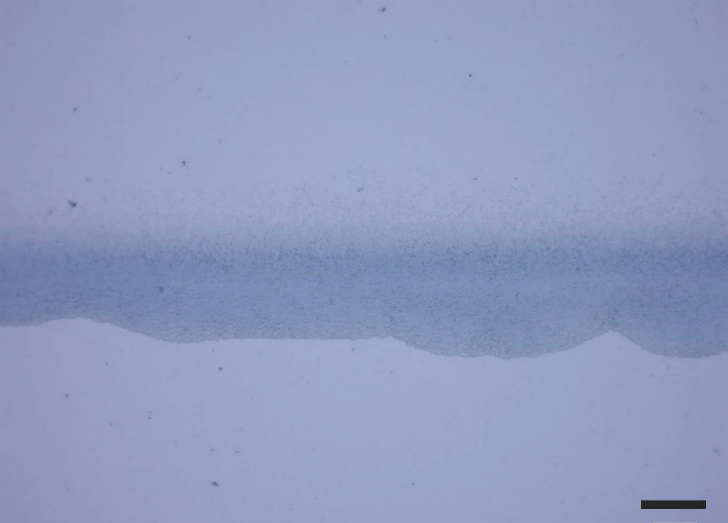


**Figure S6.** Effect of 20% v/v of ethylene glycol on the printed line. Scale bar 50 µm.


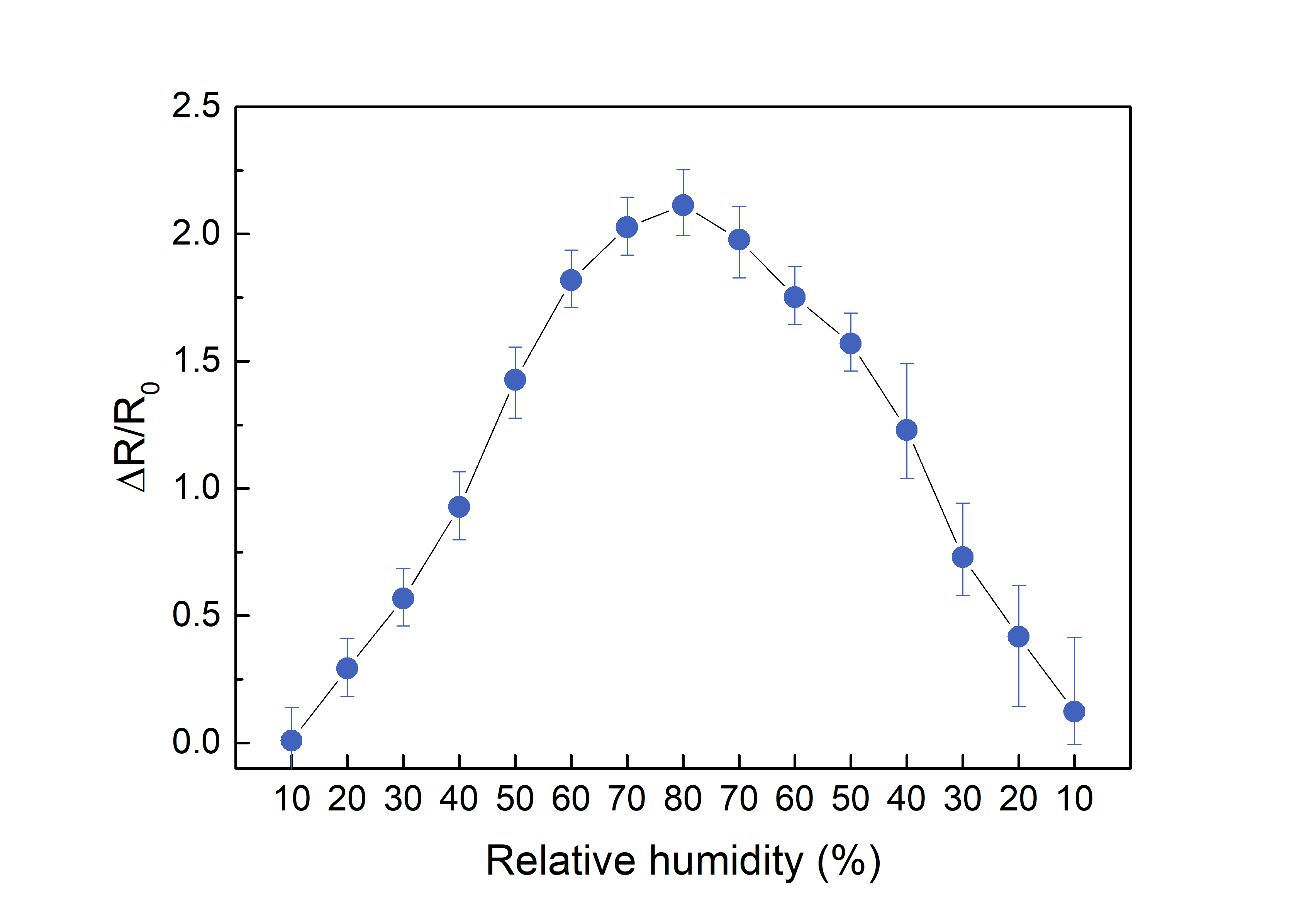


**Figure S7.** The dynamic sensor response at different RH of the PEDOT:PSS printed onto cellulose substrate.


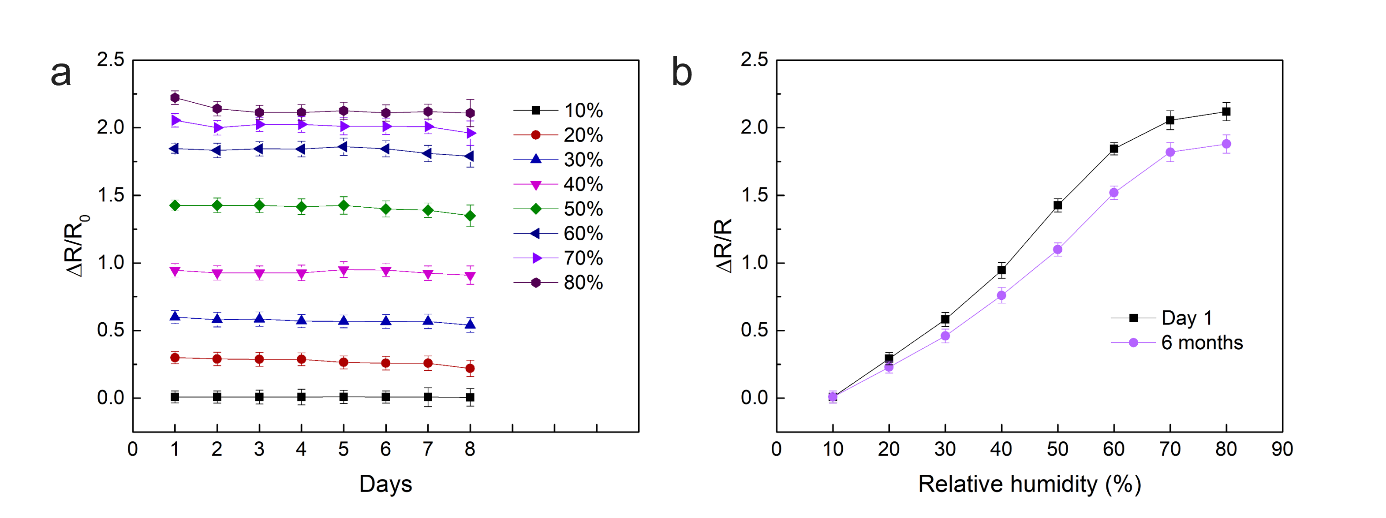


**Figure S8.** a) Stability test of the printed PEDOT:PSS humidity sensor over an 8-day period. b) Comparative stability performance of the printed PEDOT:PSS humidity sensor on day one and after six months.

**Supplementary References**

[1] X. Aeby, J. Bourely, A. Poulin, G. Siqueira, G. Nyström, and D. Briand, “Printed Humidity Sensors from Renewable and Biodegradable Materials,” *Advanced Materials Technologies*, vol. 8, no. 5, p. 2201302, Mar. 2023, doi: 10.1002/admt.202201302.

[2] Y.-F. Wang *et al.*, “Fully Printed PEDOT:PSS-based Temperature Sensor with High Humidity Stability for Wireless Healthcare Monitoring,” *Scientific Reports*, vol. 10, no. 1, p. 2467, Feb. 2020, doi: 10.1038/s41598-020-59432-2.

[3] A. S. G. Reddy, B. B. Narakathu, M. Z. Atashbar, M. Rebros, E. Rebrosova, and M. K. Joyce, “Fully Printed Flexible Humidity Sensor,” *Procedia Engineering*, vol. 25, pp. 120–123, Jan. 2011, doi: 10.1016/j.proeng.2011.12.030.

[4] X. Zhang *et al.*, “Printed Carbon Nanotubes-Based Flexible Resistive Humidity Sensor,” *IEEE Sensors Journal*, vol. 20, no. 21, pp. 12592–12601, Nov. 2020, doi: 10.1109/JSEN.2020.3002951.

[5] P. Zhu, H. Ou, Y. Kuang, L. Hao, J. Diao, and G. Chen, “Cellulose Nanofiber/Carbon Nanotube Dual Network-Enabled Humidity Sensor with High Sensitivity and Durability,” *ACS Appl. Mater. Interfaces*, vol. 12, no. 29, pp. 33229–33238, Jul. 2020, doi: 10.1021/acsami.0c07995.

[6] S. Tachibana *et al.*, “A Printed Flexible Humidity Sensor with High Sensitivity and Fast Response Using a Cellulose Nanofiber/Carbon Black Composite,” *ACS Appl. Mater. Interfaces*, vol. 14, no. 4, pp. 5721–5728, Feb. 2022, doi: 10.1021/acsami.1c20918.

[7] D. Zhang, H. Chang, P. Li, R. Liu, and Q. Xue, “Fabrication and characterization of an ultrasensitive humidity sensor based on metal oxide/graphene hybrid nanocomposite,” *Sensors and Actuators B: Chemical*, vol. 225, pp. 233–240, Mar. 2016, doi: 10.1016/j.snb.2015.11.024.

[8] R. A. Shaukat, M. U. Khan, Q. M. Saqib, M. Y. Chougale, J. Kim, and J. Bae, “All range highly linear and sensitive humidity sensor based on 2D material TiSi2 for real-time monitoring,” *Sensors and Actuators B: Chemical*, vol. 345, p. 130371, Oct. 2021, doi: 10.1016/j.snb.2021.130371.

[9] K. O. Babaremu *et al.*, “Sustainable plastic waste management in a circular economy,” *Heliyon*, vol. 8, no. 7, p. e09984, Jul. 2022, doi: 10.1016/j.heliyon.2022.e09984.

[10] O. US EPA, “Impacts of Plastic Pollution.” Accessed: Oct. 08, 2024. [Online]. Available: https://www.epa.gov/plastics/impacts-plastic-pollution

[11] A. Chamas *et al.*, “Degradation Rates of Plastics in the Environment,” *ACS Sustainable Chem. Eng.*, vol. 8, no. 9, pp. 3494–3511, Mar. 2020, doi: 10.1021/acssuschemeng.9b06635.

[12] E. Rezvani Ghomi *et al.*, “The Life Cycle Assessment for Polylactic Acid (PLA) to Make It a Low-Carbon Material,” *Polymers*, vol. 13, no. 11, 2021, doi: 10.3390/polym13111854.

[13] K. W. Meereboer, M. Misra, and A. K. Mohanty, “Review of recent advances in the biodegradability of polyhydroxyalkanoate (PHA) bioplastics and their composites,” *Green Chem.*, vol. 22, no. 17, pp. 5519–5558, 2020, doi: 10.1039/D0GC01647K.

[14] “Cellulose Di-Acetat from Bleher film technology.” Accessed: Oct. 08, 2024. [Online]. Available: https://www.bleher.com/en/cellulose-di-acetat/

[15] E. Wawrzynek, C. Baumbauer, and A. C. Arias, “Characterization and Comparison of Biodegradable Printed Capacitive Humidity Sensors,” *Sensors*, vol. 21, no. 19, 2021, doi: 10.3390/s21196557.

[16] C. Gaspar, J. Olkkonen, S. Passoja, and M. Smolander, “Paper as Active Layer in Inkjet-Printed Capacitive Humidity Sensors,” *Sensors*, vol. 17, no. 7, 2017, doi: 10.3390/s17071464.

[17] N. M. Pereira *et al.*, “Aerosol-Printed MoS2 Ink as a High Sensitivity Humidity Sensor,” *ACS Omega*, vol. 7, no. 11, pp. 9388–9396, Mar. 2022, doi: 10.1021/acsomega.1c06525.
